# A Comprehensive Analysis of Potassium Bromate, a Possible Carcinogen, in Popular Baked Foodstuffs of Bangladesh

**DOI:** 10.1002/fsn3.4546

**Published:** 2024-10-24

**Authors:** Md. Mazharul Islam, Supath Xavier Besra, Shahnaz Akhtar Nishat, Abida Sultana

**Affiliations:** ^1^ Department of Chemistry University of Dhaka Dhaka Bangladesh

**Keywords:** baked foodstuffs, carcinogenicity, potassium bromate, regulatory compliance, UV–visible spectrophotometer

## Abstract

Potassium bromate (KBrO_3_), employed ubiquitously as an oxidizing agent in commonly consumed baked foods and as a flour improver to enhance dough quality, has been recognized as a possible human carcinogen. Despite stringent prohibitions in numerous nations eliciting substantial apprehension worldwide, notably in Bangladesh, the pervasive occurrence of KBrO_3_ in baked foods persists as a formidable public health quandary. This study aimed to investigate the presence of KBrO_3_ in five different popular baked foods comprising a total of 104 samples, such as bread (*n* = 39), cake (*n* = 33), burger buns (*n* = 13), pizza (*n* = 10), and naan (*n* = 9), collected from different districts across Bangladesh. The amounts of KBrO_3_ were quantified using UV–visible spectrophotometric method. A calibration curve was obtained with a satisfactory linearity and a correlation coefficient (*R* ^2^) 0.998. The sensitivity was confirmed by limits of detection (LOD = 0.0180 mg L^−1^) and quantification (LOQ = 0.0548 mg L^−1^). The recovery rates for spiked samples were 87%–103%, ensuring the accuracy and reliability of the measurements. KBrO_3_ concentrations varied significantly across the samples: BDL to 5.53 ± 0.24 mg kg^−1^ in breads, BDL to 6.18 ± 0.01 mg kg^−1^ in cakes, 0.86 ± 0.01 to 1.69 ± 0.01 mg kg^−1^ in burger buns, 0.92 ± 0.01 to 4.35 ± 0.01 mg kg^−1^ in pizzas, and BDL to 5.37 ± 0.02 mg kg^−1^ in naans. The concentrations of KBrO_3_ in bread, cake, burger buns, pizza, and naans surpass the recommended threshold established by the FDA factors of 276.5, 309.0, 84.5, 217.5, and 268.5, respectively. The study revealed particularly high levels of KBrO_3_ presence in the targeted baked foods in all of the sampling districts, highlighting a serious food safety concern in Bangladesh.

## Graphical abstract

This study aimed to investigate the presence of KBrO_3_ in five different popular baked foods comprising a total of 104 samples. The study revealed particularly high levels of KBrO_3_ presence in the targeted baked foods in all the sampling districts, highlighting a serious food safety concern in Bangladesh.

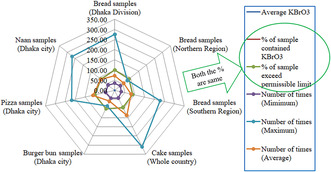

## 1 Introduction

Baked products like bread, cake, burger buns, pizza, and naan are favored by people of all ages in developing countries, particularly Bangladesh, for their nutritional value and easy accessibility. They serve as convenient snacks or breakfast options, meeting the needs of busy individuals seeking quick and satisfying meals. The popularity of these ready‐to‐eat foods is driven by factors such as urbanization and greater female workforce participation (Horning et al. 2017). Studies have highlighted their role in addressing minor hunger pangs (Hossain et al. 2023) and providing a convenient food solution for consumers on the go. This has led to a growing demand for baked foods offering extended shelf life, an appealing taste, portability, and nutritional value, typically comprising flour, leavening agents, salts, yeast, and water, with potential additions such as eggs, sugar, meat, shortening, and other chemicals based on desired characteristics and taste on the products (Amani et al. 2021; Mahmud et al. 2021). The bakery industry in Bangladesh is witnessing substantial growth, driven by evolving consumer tastes and increasing purchasing power, with the bread market projected to reach 4.51 billion kg by 2028, showing a 6.3% volume growth by 2024 (Akij Bakers Limited (ABL) 2023).

Potassium bromate (KBrO_3_) is an odorless, colorless, and tasteless white crystalline oxidizing agent utilized in various industrial applications, notably employed in the food sector as an alluring food additive (Yalçin and Çavuşoğlu 2022). For over 90 years, KBrO_3_ has been widely used as a flour additive due to its ability to improve dough elasticity (Oloyede and Sunmonu 2009), retain CO_2_ during leavening (Hama 2022), and enhance bread texture and volume (Magomya and Yebpella 2020). It also improves elasticity, cohesion, swelling ability, and visual appeal, resulting in a fluffy and soft finished product (Emeje et al. 2015; Naze, Epete, and Owhoeke 2018). It is likely the most cost‐effective oxidizer in the fermentation and proofing phase for bread production (Uwah and Ikwebe 2020), but excessive use can leave harmful residues in the final product. During baking, heat converts KBrO_3_ to the less toxic KBr (Ati‐Hellal et al. 2018), yet residual concentrations can still pose health risks (Ncheuveu Nkwatoh, Fon, and Navti 2023). It is recognized as a potential and probable human carcinogen (IARC 1986). Consequently, numerous organizations and countries have prohibited its use as a flour additive (Chauhan and Jain 2016; FAO/WHO JECFA 1992). KBrO_3_ in the bloodstream can generate highly reactive compounds, posing a risk of DNA damage and contributing to cancer formation, as supported by studies on animal models like rats (Magomya and Yebpella 2020; Olusola et al. 2024). Numerous literature findings document the cytotoxic, genotoxic, mutagenic, carcinogenic, and biochemical adverse effects associated with KBrO_3_ exposure (Zhang et al. 2011). Acutely, humans may experience symptoms such as nausea, vomiting, diarrhea, and abdominal pain, while chronic exposure may lead to conditions including reduced urine output, deafness, vertigo, low blood pressure, neurological impairment, kidney dysfunction, and decreased platelet count (Ajarem et al. 2016; Shanmugavel et al. 2020). Apart from its carcinogenic properties, KBrO_3_ has been observed to affect the nutritional quality of bread, leading to a depletion of essential vitamins such as vitamin A2, B1, B2, and niacin when used excessively (Okafor, Ezeonu, and Ogbodo 2011).

This research aims to comprehensively estimate the presence of the possible carcinogen KBrO_3_ in various baked goods (bread, cake, burger bun, pizza, and naan) available in Dhaka, its surrounding cities and other prominent divisional cities, and districts in Bangladesh by using UV–visible spectrophotometric methods. The study intends to validate an established analytical method to accurately quantify KBrO_3_ residues in finished baked products. This research provides critical data that can inform regulatory authorities and prompt necessary actions to mitigate the use of this harmful additive in the food industry. Ultimately, the objective is to ensure enhanced food safety and protect public health in Bangladesh by establishing a reliable methodology for monitoring KBrO_3_ levels in bread, cake, burger bun, pizza, and naan samples.

## 2 Experimental

### 2.1 Solvents and Reagents

Chemicals of analytical grade purity and distilled deionized water were used throughout the experimental work. Potassium bromate (Smart lab, Indonesia), 37% Hydrochloric acid (BDH, UK), and promethazine hydrochloride (Harika Drugs Private Limited, Hyderabad, India) were used for bromate determination. Solvents such as n‐hexane (RCI Labscan Limited, Bangkok, Thailand) and ethyl acetate (RCI Labscan Limited, Bangkok, Thailand) were utilized for extraction processes at different levels of the experiment. Sulfuric acid (95%–97%, Merck, Germany) and Potassium bromide (Merck, Germany) were both used in this research work. Sodium Chloride, anhydrous Sodium sulfate, and Sodium thiosulfate were acquired from Scharlau, Germany.

### 2.2 Instruments

All the investigations were conducted with the aid of an oven and furnace (GSM 11/8 Hope Valley, S336RB, England), kitchen blender (Miyako Chopper, Japan), an analytical balance (model‐ AL 104, Company‐ Mettler Toledo, US), an electric balance (FR‐200, NDO‐450ND, Japan), a rotary vacuum evaporator (Heidolph, Germany), a centrifuge machine (Hanil Science Industrial Co. Ltd., Model‐Combi 514 R) with rotation up to 4000 rpm, a double beam UV spectrophotometer (Model: UV‐1800, Shimadzu), and a vortex machine (Cat/Art No. 444‐1372 (EU), Made in Germany).

### 2.3 Sampling Strategy

In total, 104 samples were collected from various bakeries, restaurants, supershops, and fast‐food places in Dhaka and different districts of Bangladesh. There were 39 breads, 33 cakes, 13 burger buns, 10 pizzas, and 9 naan samples as these baked products are now getting popular to this modern hectic daily life of people of all classes (Islam and Ullah 2010; Shifat, Shibli, and Islam 2023). The choice of these samples was based on the highest consumption and popularity among the populace in each location (Dudziak, Stoma, and Osmólska 2023). The samples were coded as bread samples (B‐1 to B‐39 in Table 2), cake samples (C‐1 to C‐33 in Table 3), burger bun samples (BB‐1 to BB‐13 in Table 4), pizza samples (P‐1 to P‐10 in Table 5) and naan samples (N‐1 to N‐9 in Table 5). The samples were gathered at the beginning of the day and immediately taken to the laboratory for analysis. All samples were acquired and analyzed within the recommended time of consumption (Compaore et al. 2022).

### 2.4 Sample Pretreatment

From the center of each sample type, approximately 10–12 g was obtained and dried at 75°C in an oven for about 2 h (Irogbeyi et al. 2019; Dagari et al. 2022). Due to the greasy nature of the cakes and the presence of cheese, meats, and various other toppings in the case of pizza, the final extract for spectrophotometric analysis was rendered turbid. Hence, a defatting process was required to remove the cloudiness of the final extract, which was achieved by heating the samples with n‐hexane in a water bath (Hua et al. 2020). The defatted samples were then dried in an oven. All the dried samples were then pulverized in a grinder into finely powdered samples, put in zip‐lock bags, and stored in a refrigerator for further analysis.

### 2.5 Preparation of Standard Solution

**FIGURE 1:**
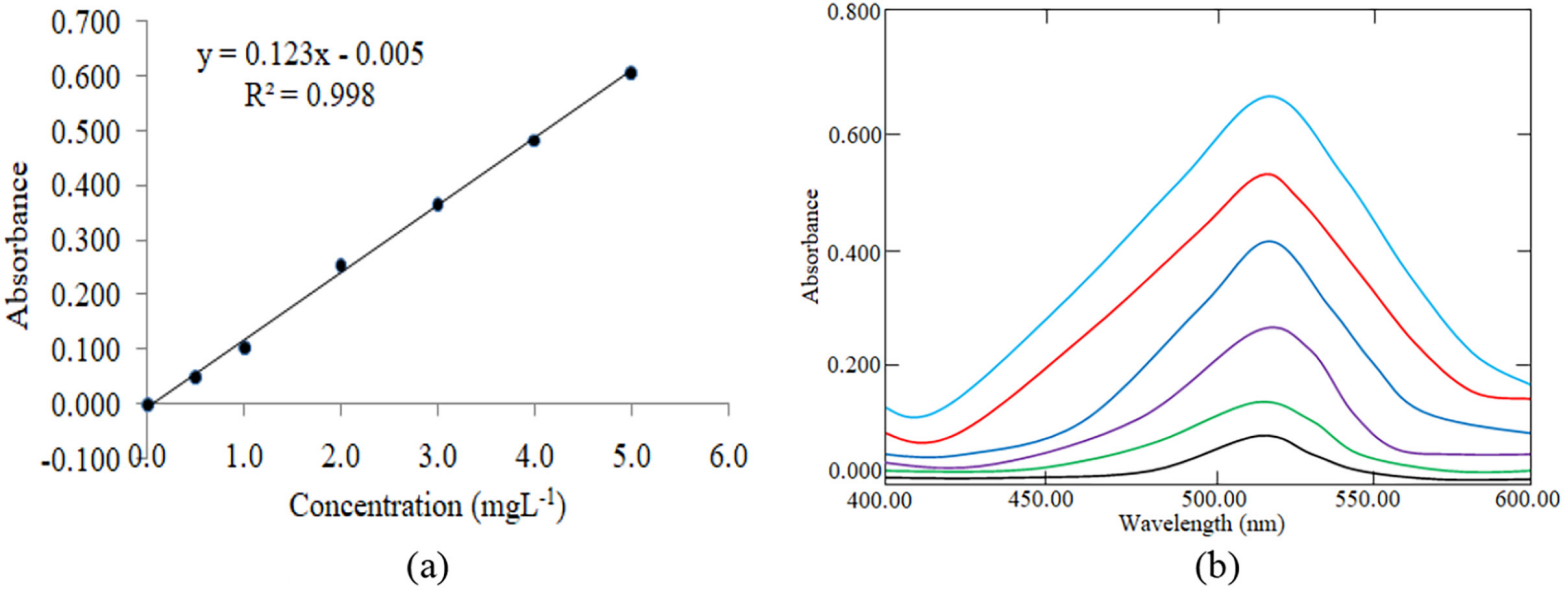
Calibration curve (a) and the overlain spectrum of KBrO3 (b).

The primary stock solution of concentration 50 mg L^−1^ was prepared by dissolving 0.0025 g of KBrO_3_ in a 50.0 mL volumetric flask with distilled water. Aliquots of 100, 200, 400, 600, 800, and 1000 μL from the primary stock solution were taken in individual 10.0 mL volumetric flasks to which 1.0 mL of 0.01 M promethazine (PMZ) and 0.2 M of 12 M HCl were added. The mixtures were then diluted up to the 10.0 mL mark of the volumetric flasks to obtain the final concentrations of the potassium bromate in the range of 0.5 to 5.0 mg L^−1^. The solutions were well shaken in an ultrasonicator for about 1 min and the absorbance was measured at 515 nm (*λ*_max_) against a reagent blank (El‐Harti et al. 2011; Mahmud et al. 2021). The results obtained were used to plot the calibration curve (Figure 1a) and the regression equation of the calibration plot was calculated by the least squares method for further determination of the amount of potassium bromate in the samples. An overlain spectrum of the prepared standard solution is shown in Figure 1b to show the different absorption at different concentrations.

### 2.6 Method Validation

The method validation was conducted through the analysis of KBrO_3_ standard solutions within a range of 0.5–5.0 mg L^−1^. A calibration curve was obtained with satisfactory linearity with an acceptable range: *R* ^2^ > 0.8 for chemistry (Chicco, Warrens, and Jurman 2021), correlation coefficient (*R* ^2^) of 0.998 (Alzeer et al. 2023). The sensitivity of the instruments was performed by the LOD (LOD = 0.0180 mg L^−1^) and LOQ (LOQ = 0.0548 μg/mL). The LOD and LOQ were calculated using the following equations (Apostol et al. 2009):

$$\mathrm{LOD}=\frac{3.3\;\sigma }{b}\;\text{and}\;\mathrm{LOQ}=\frac{10\;\sigma }{b}$$

where *σ* = standard deviation of intercept of the plot of absorbance versus bromate concentration of the samples, *b* = slope of the plot of absorbance versus bromate concentration of the samples. The accuracy was tested by calculating the recovery of different bakery samples spiked with known amounts of potassium bromated (Xu et al. 2012; Naveen et al. 2018). A recovery analysis was conducted, which included five different samples (B‐1, C‐12, BB‐5, P‐5 and N‐2) for spiking at 1.25 mg kg^−1^ to assess the accuracy and precision of the current method employed for the determination of bromate. To 1 g of each sample measured 500 μL of 50 mg L^−1^ of bromate was added to spike the samples (Sima et al. 2024). For about 10 min, the sample matrixes were left to stand for mixing. As previously described method (El‐Harti et al. 2011), bromate analysis was performed on each spiked sample. The following formula was utilized for the determination of the recovery (*R*) percentage.

$$R\;\left(\%\right)=\frac{\text{Amount of bromate in the spiked sample}-\text{Amount of bromate in the unspiked sample}}{\text{Added amount of bromate}}\times 100$$

### 2.7 Sample Analysis

**FIGURE 2:**
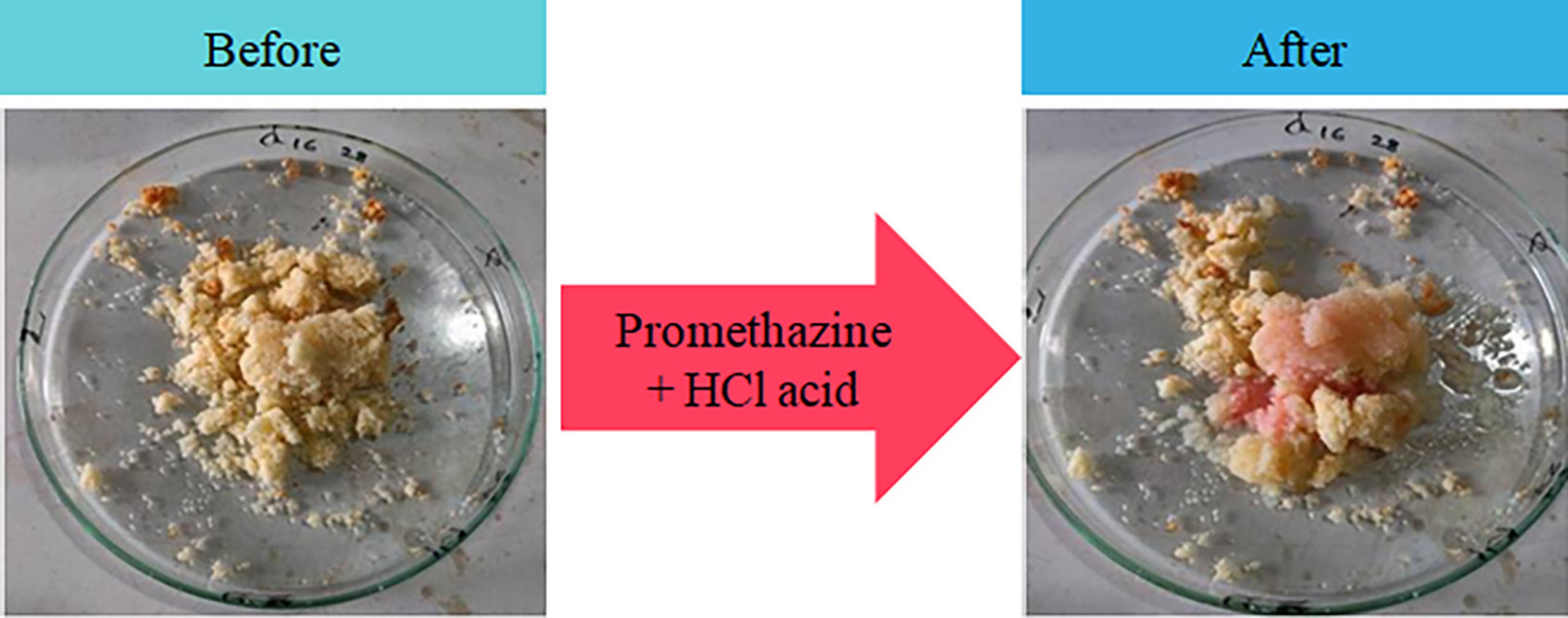
Preliminary tests: Before and after adding PMZ and hydrochloric acid.

A preliminary confirmative test was performed directly on a portion of the sample with 2.0 mL of 0.01 M PMZ and 0.6 mL of 12 M hydrochloric acid (Rahil and Sharaa 2023). The change in color of each sample to pink suggests the presence of KBrO_3_ (Figure 2).

**FIGURE 3:**
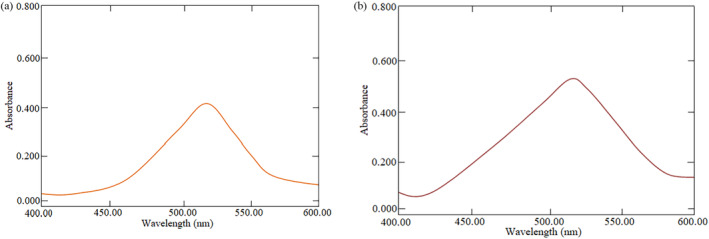
Sample spectra (a) Sample P‐4 and (b) Sample C‐29.

Quantitative determination of potassium bromate in the samples was carried out according to spectrophotometric method described by El‐Harti et al. (2011). From the previously pulverized samples stored in zip‐lock bags, 1.0 g of the finely powdered sample was weighed into a conical flask to which 20 mL of distilled water was added for the extraction of potassium bromate from the sample. The mixture was shaken thoroughly with the aid of a vortex machine and then filtered using a Whatman no.1 filter paper. The filtrate (8.0 mL) was transferred into a 10.0 mL volumetric flask to which 2.0 mL of 0.01 M PMZ followed by 0.20 mL of 12 M hydrochloric acid were added. The mixture was shaken well for about a minute and the absorbance of the resulting solution was measured using a double‐ beam UV‐ spectrophotometer at 515 nm (Mahmud et al. 2021) (Figure 3). The concentration of potassium bromate in the sample was derived from the linear regression curve obtained from the standard solutions of potassium bromate as mentioned above.

## 3 Results and Discussion

The method validation was performed by the analysis of KBrO_3_ calibration solutions within 0.5–5.0 mg L^−1^ (Ati‐Hellal et al. 2018). A calibration curve was obtained with a satisfactory linearity with a correlation coefficient (*R* ^2^) of 0.998. The sensitivity of the instrument was confirmed by the LOD (LOD = 0.0180 mg L^−1^) and LOQ (LOQ = 0.0548 mg L^−1^). The method showed good recoveries ranging from 87%–103% (Table 1) indicating the excellent accuracy and precision of the sample analysis method.

**TABLE 1:** Method validation parameters for the bromated determination in baked products.

Parameters	Value	Comments
Wavelength (*λ*_max_)	515 nm	UV–visible spectrophotometer
Regression equation	*y* = 0.123*x*–0.005	Linear equation
Linearity	0.5–5.0 mg kg^−1^	For calibration
*R* ^2^	0.998	In acceptable range
LOD	0.0180 mg kg^−1^	Minimum detectable concentration
LOQ	0.0548 mg kg^−1^	Minimum quantifiable concentration
Recovery: Spiking level 1.25 mg kg^−1^	Recovery: 87%–103%	Five replicates ware done for this spiking level

The presence of bromate in bread samples from different regions of Bangladesh (Table 2) reveals significant variability in contamination levels, with some areas showing notably higher concentrations than others (Olusola et al. 2024). Bromate levels in bread samples ranged from below detection limits (BDLs) to 5.53 mg kg^−1^, indicating varying baking practices across regions. In the Dhaka Division, the highest bromate concentration was in Rajbari (5.53 mg kg^−1^, sample B‐14), which significantly exceeds the other samples from this division. This high level raises concerns about the potential health risks associated with bromate, a known carcinogen (Ayembilla et al. 2024). Similarly, Mohammadpur (3.46 mg kg^−1^, sample B‐4) and Tangail (2.56 mg kg^−1^, sample B‐17) also showed elevated levels, suggesting localized contamination or specific baking additives that include potassium bromate.

**TABLE 2:** Bromate in bread samples (*n* = 39) collected from different regions of Bangladesh.

Bread sample	Sampling date	Location	Bromate (mg kg^−1^)	RSD (%)	Bread sample	Sampling date	Location	Bromate (mg kg^−1^)	RSD (%)
**Dhaka division (B‐1 to B‐19)**									
B‐1	20.04.2022	Mirpur	1.35 ± 0.01	0.39	B‐11	23.05.2022	Dhanmondi	1.01 ± 0.03	2.91
B‐2	21.04.2022	Lalbagh	0.77 ± 0.02	2.44	B‐12	26.05.2022	Gazipur	0.90 ± 0.03	2.97
B‐3	24.04.2022	Jatrabari	0.96 ± 0.01	0.55	B‐13	29.05.2022	Narsingdi	0.71 ± 0.01	1.12
B‐4	25.04.2022	Mohammadpur	3.46 ± 0.01	0.31	B‐14	29.05.2022	Rajbari	5.53 ± 0.24	4.37
B‐5	28.04.2022	Tongi	1.08 ± 0.01	0.49	B‐15	30.05.2022	Narayanganj	0.81 ± 0.02	2.30
B‐6	15.05.2022	Tejgaon	0.92 ± 0.01	0.58	B‐16	31.05.2022	Munshiganj	2.01 ± 0.01	0.80
B‐7	15.05.2022	Uttara	0.83 ± 0.02	2.60	B‐17	24.05.2022	Tangail	2.56 ± 0.01	0.21
B‐8	16.05.2022	Motijheel	0.82 ± 0.01	1.31	B‐18	27.05.2022	Faridpur	0.70 ± 0.01	1.15
B‐9	19.05.2022	Gulshan	1.15 ± 0.01	0.84	B‐19	28.05.2022	Madaripur	0.75 ± 0.01	1.06
B‐10	22.05.2022	Akran, Savar	1.23 ± 0.04	3.06	—	—	—	—	—
**Northern Region of Bangladesh (B‐20 to B‐29)**					**Southern Region of Bangladesh (B‐30 to B‐39)**				
B‐20	24.05.2022	Mymensingh	0.77 ± 0.03	4.01	B‐30	15.06.2022	Jessore	0.74 ± 0.03	4.57
B‐21	01.06.2022	Sylhet	0.72 ± 0.01	0.43	B‐31	16.06.2022	Noakhali	4.56 ± 0.31	6.86
B‐22	06.06.2022	Dinajpur	0.95 ± 0.01	0.64	B‐32	19.06.2022	Patuakhali	4.51 ± 0.22	4.95
B‐23	29.06.2022	Panchagarh	0.80 ± 0.01	1.54	B‐33	21.06.2022	Chattogram	0.95 ± 0.07	7.79
B‐24	10.07.2022	Pabna	0.93 ± 0.01	1.00	B‐34	30.06.2022	Barisal	0.96 ± 0.02	2.91
B‐25	18.07.2022	Kishoreganj	1.21 ± 0.01	1.02	B‐35	01.07.2022	Cumilla	0.79 ± 0.01	0.77
B‐26	21.07.2022	Rajshahi	BDL	—	B‐36	02.07.2022	Khulna	BDL	—
B‐27	27.07.2022	Bogra	1.74 ± 0.01	0.89	B‐37	29.07.2022	Bandarban	0.71 ± 0.01	1.74
B‐28	31.07.2022	Tangail	1.95 ± 0.01	0.47	B‐38	12.08.2022	Bagerhat	0.66 ± 0.01	1.88
B‐29	08.08.2022	Joypurhat	1.14 ± 0.01	0.27	B‐39	13.08.2022	Bhola	0.96 ± 0.13	14.55

*Note:* Values are given as the mean ± SD of three measurements. BDL is the below detection limit.

**FIGURE 4:**
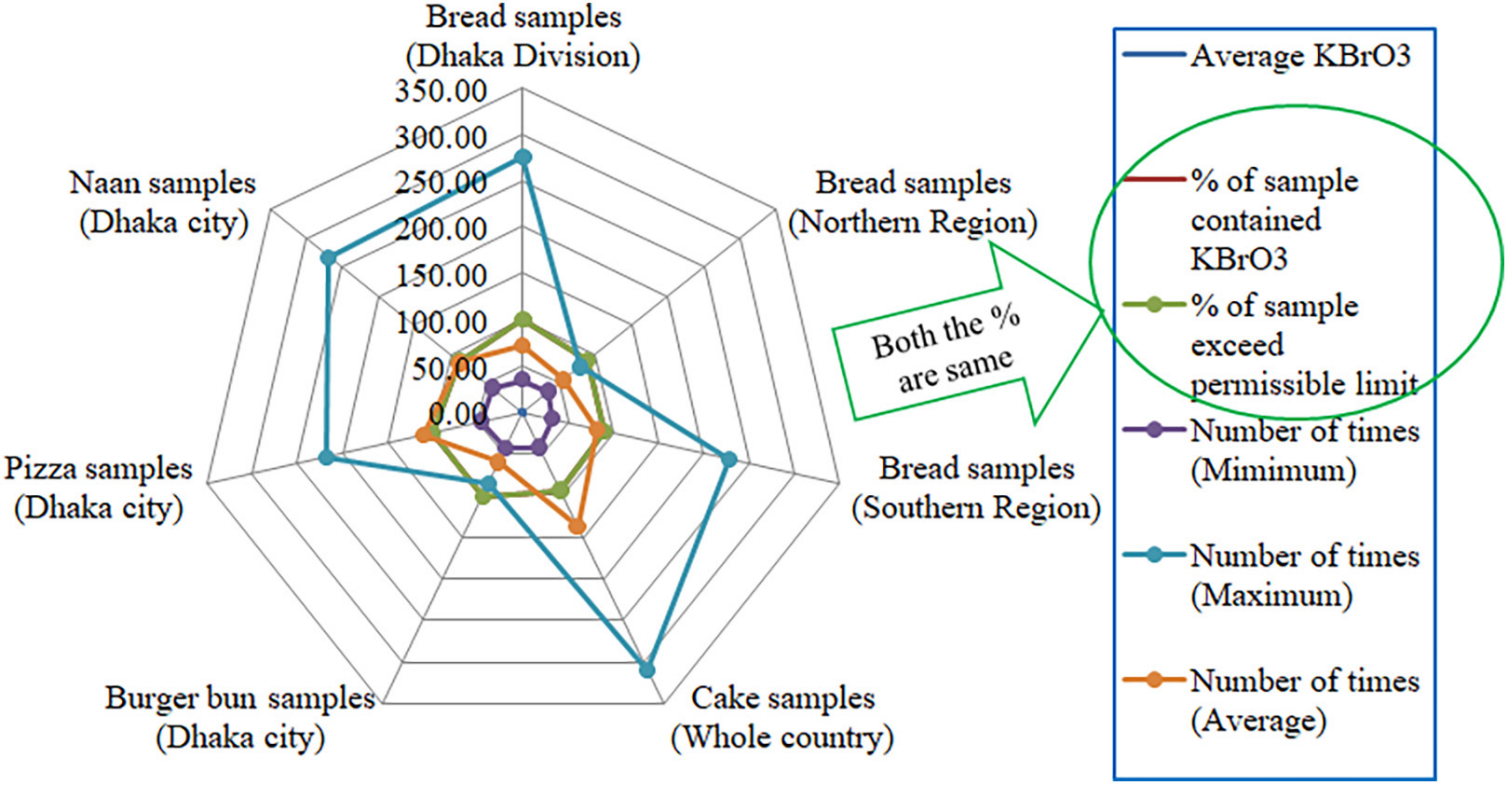
Overview of bromate content in baked goods across the country.

The samples of the Northern Region of Bangladesh exhibited generally lower bromate levels, except Bogra (1.74 mg kg^−1^, sample B‐27) and Tangail (1.95 mg kg^−1^, sample B‐28). Interestingly, Rajshahi and Khulna samples (B‐26 and B‐36) were BDL, which might reflect different regulatory practices or the use of bromate‐free baking agents in these areas. In the samples of the Southern Region of Bangladesh, significant bromate levels were found in Noakhali (4.56 mg kg^−1^, sample B‐31) and Patuakhali (4.51 mg kg^−1^, sample B‐32). The variability in bromate levels here, ranging from BDL to 4.56 mg kg^−1^, suggests inconsistent adherence to food safety regulations or variable sourcing of baking ingredients (Ayembilla et al. 2024). The relative standard deviations (RSDs) across the samples indicate the precision of the measurements, with most samples showing RSDs below 5%, except for a few outliers like Bhola (14.55%, sample B‐39) suggesting an inhomogeneous distribution of bromate within the sample or variations in the sample preparation process (Coleman 2012). In Dhaka Division, 100% of samples exceeded the FDA's permissible limit of 0.02 mg kg^−1^ (American Bakers Association and American Institute of Baking International (ABA/AIBI) 2008; Ekop, Obot, and Ikpatt 2008), with an average concentration of 1.45 mg kg^−1^, up to 276.5 times the permissible level. The Northern Region had 90% of samples exceeding the limit, averaging 1.33 mg kg^−1^, up to 79.5 times the limit. The Southern Region also had 90% of samples above the limit, with an average of 1.65 mg kg^−1^, up to 228 times the limit (Figure 4). These findings indicate significant health risks, necessitating urgent regulatory action. Overall, these findings underscore the need for stringent monitoring and regulation of bromate in bread to ensure food safety across Bangladesh. Mahmud et al., conducted a spectrophotometric analysis of 21 collected bread samples from bakeries and shops in and around Dhaka City revealing that 67% had KBrO_3_ levels exceeding global FDA permissible limits, with the highest concentration detected at 9.29 mg kg^−1^ (Mahmud et al. 2021). But in this study, 94% sample exceeded the permissible limit and most of the samples contained KBrO_3_ much less than that of Mahmud et al. One of our recent studies revealed that, out of 30 randomly collected bread samples (including buns, white bread, and sliced bread) from various parts of Dhaka city, potassium bromate levels ranged from 1.24 to 24.91 mg kg^−1^. It was found that 73% of these samples contained potassium bromate levels exceeding the FDA's permissible safe limit for bread (Sima et al. 2024). Another study analyzing bread samples found potassium bromate levels ranging from 0.0001 to 12.16 μg/g; out of 210 samples, 119 contained potassium bromate, with 35 exceeding the FDA limit and 84 below the safe threshold (Dagari et al. 2022). Rana et al. analyzed 14 bread samples from various brands from bakeries, retailers, and fast‐food outlets in Jaipur, Rajasthan, India, with the fruit bun from brand I exhibiting the highest concentration (39.73 mg kg^−1^) of potassium bromate and the white bread from brand III showing the lowest (10.72 mg kg^−1^) (Rana, Chaturvedi, and Sharma 2020). Ekere et al., assessed bread samples, randomly collected from 10 different vendors in the Jos metropolis, which exhibited potassium bromate ranging from 250 to 4375 mg/kg, with a mean concentration significantly exceeding the maximum permissible level of 0.02 mg kg^−1^ (Ekere et al. 2020). Nkwatoh et al. collected 31 samples of popular bread types from bakeries and vendors in Bamenda, North West Region of Cameroon, finding potassium bromate concentrations in all samples (100%) ranging from 48.50 mg kg^−1^ to 10,148.50 mg kg^−1^, which exceeded the FDA's maximum acceptable limit of 50 mg kg^−1^ by 9–203 times (Ncheuveu Nkwatoh, Fon, and Navti 2023).

The bromate levels in cake samples from different regions of Bangladesh revealed a broad range of concentrations (Table 3). Among the 33 samples, the highest bromate concentration was detected in a cake from Barishal (6.18 mg kg^−1^, sample C‐22), while the lowest detectable concentration was in Lalmatia (0.84 mg kg^−1^, sample C‐11). Two samples (from Mohammadpur and Jessore) had bromate levels BDL. The RSD values ranged from 0.79% to 17.80%, indicating varying levels of precision in the measurements. High RSD values in samples from Mymensingh (9.95%), Lalmatia (11.08%), and Bandarbaan (10.21%) suggest potential inconsistencies in bromate distribution within these cakes or variations in the analytical procedure. The widespread presence of bromate, a potential carcinogen, in cakes across many regions underscores the need for stringent regulatory measures and consistent monitoring to ensure food safety. The analysis of cake samples across the country revealed an average potassium bromate concentration of 2.74 mg kg^−1^. Potassium bromate was detected in 93% of the samples, all of which exceeded the FDA's permissible limit of 0.02 mg kg^−1^. The contamination levels ranged from 42 to 309 times the permissible limit, with an average exceedance of 137.21 times (Figure 4). This widespread and significant contamination highlights a serious public health concern requiring immediate regulatory attention.

**TABLE 3:** Bromate in cake samples (*n* = 33) collected from different regions of Bangladesh.

Cake sample	Sampling date	Location	Bromate (mg kg^−1^)	RSD (%)	Cake sample	Sampling date	Location	Bromate (mg kg^−1^)	RSD (%)
C‐1	13.04.22	Shahbag	2.06 ± 0.01	2.15	C‐18	20.06.22	Tejgaon	2.36 ± 0.01	3.05
C‐2	18.04.22	Khilgaon	1.71 ± 0.01	3.05	C‐19	26.06.22	Joypurhaat	2.85 ± 0.01	2.08
C‐3	20.04.22	Chankharpool	4.66 ± 0.01	1.14	C‐20	29.06.22	Pabna	2.51 ± 0.01	1.06
C‐4	21.04.22	Azimpur	3.14 ± 0.02	7.18	C‐21	05.07.22	Panchagarh	2.39 ± 0.01	6.04
C‐5	24.04.22	Mymensingh	1.09 ± 0.01	9.95	C‐22	13.07.22	Barishal	6.18 ± 0.01	1.34
C‐6	27.04.22	Jatrabari	3.15 ± 0.02	6.40	C‐23	17.07.22	Cumilla	4.52 ± 0.01	1.33
C‐7	08.05.22	Gazipur	2.01 ± 0.01	2.46	C‐24	20.07.22	Jessore	BDL	—
C‐8	11.05.22	Mirpur	2.65 ± 0.01	1.57	C‐25	24.07.22	Narsingdi	2.98 ± 0.01	2.70
C‐9	12.05.22	Mohammadpur	BDL	—	C‐26	27.07.22	Rajshahi	2.26 ± 0.01	3.42
C‐10	16.05.22	Dhanmondi	3.78 ± 0.01	3.10	C‐27	01.08.22	Kishoreganj	1.81 ± 0.01	5.55
C‐11	19.05.22	Lalmatia	0.84 ± 0.01	11.08	C‐28	04.08.22	Bandarbaan	1.19 ± 0.01	10.21
C‐12	24.05.22	Shyamoli	1.23 ± 0.01	2.49	C‐29	08.08.22	Chattogram	4.62 ± 0.01	1.96
C‐13	29.05.22	Savar	1.30 ± 0.01	5.58	C‐30	11.08.22	Motijheel	1.06 ± 0.01	17.80
C‐14	02.06.22	Patuakhali	3.21 ± 0.01	0.79	C‐31	14.08.22	Narayanganj	4.09 ± 0.01	2.60
C‐15	07.06.22	Rajbari	2.70 ± 0.01	2.27	C‐32	18.08.22	Lalbagh	2.72 ± 0.01	4.28
C‐16	13.06.22	Khulna	4.42 ± 0.02	3.07	C‐33	22.08.22	Sylhet	1.76 ± 0.01	7.98
C‐17	16.06.22	Dinajpur	3.82 ± 0.02	3.90	—	—	—	—	—

The bromate levels in 13 burger bun samples collected from various locations in Dhaka city were analyzed, revealing concentrations ranging from 0.86 mg kg^−1^ to 1.69 mg kg^−1^ (Table 4). The highest bromate concentration was found in a sample from Dhanmondi (Road 10) at 1.69 mg kg^−1^, while the lowest was from Dhanmondi (Road 8) at 0.86 mg kg^−1^. The RSD values varied from 0.18% to 1.82%, indicating high measurement precision. Consistent bromate presence was noted across different locations, including Mirpur, Dhanmondi, and Mohammadpur, with most samples showing similar bromate levels. This suggests that bromate is widely used in burger buns in Dhaka. The low RSD values, especially for Dhanmondi (Road 10) at 0.18%, demonstrate reliable and precise analytical methods. The detection of bromate in all tested samples raises significant public health concerns due to its potential carcinogenic effects. These findings underscore the urgent need for regulatory intervention and ongoing monitoring to ensure bromate levels in bakery products are minimized.

**TABLE 4:** Bromate in burger bun samples (*n* = 13) collected from Dhaka city.

Bun Samples	Sampling date	Location	Bromate (mg kg^−1^)	RSD (%)
BB‐1	13.07.2022	Mirpur‐1	1.12 ± 0.02	1.65
BB‐2	14.07.2022	Dhanmondi (Road 8)	0.86 ± 0.01	0.36
BB‐3	23.07.2022	Mirpur‐2	0.96 ± 0.01	0.64
BB‐4	22.07.2022	Lalbagh	1.02 ± 0.01	1.21
BB‐5	24.07.2022	Azimpur	1.34 ± 0.01	0.69
BB‐6	28.07.2022	Dhanmondi (Road 10)	1.69 ± 0.01	0.18
BB‐7	01.08.2022	Khilgaon	1.34 ± 0.01	0.92
BB‐8	01.08.2022	Dhanmondi (Road 12)	1.19 ± 0.01	1.56
BB‐9	01.08.2022	Mohammadpur‐West	1.17 ± 0.01	1.05
BB‐10	05.08.2022	Mirpur‐10	1.16 ± 0.02	1.34
BB‐11	05.08.2022	Rampura	0.96 ± 0.01	0.64
BB‐12	06.08.2022	Mohammadpur‐East	1.53 ± 0.01	1.01
BB‐13	10.08.2022	Mirpur‐11	1.18 ± 0.02	1.82

Additionally, the study suggests that further research is needed to identify the sources of bromate contamination in burger buns. Implementing strategies to eliminate bromate from the food production process will be crucial to enhancing food safety. Ensuring compliance with food safety standards and raising awareness among producers and consumers about the risks associated with bromate can help mitigate these health hazards. Analysis of burger bun samples from Dhaka city showed an average bromated of 1.19 mg kg^−1^. It was present in 100% of the samples, all exceeding the FDA's permissible limit of 0.02 mg kg^−1^. The contamination ranged from 43.0 to 84.5 times the permissible limit, with an average exceedance of 59.69 times (Figure 4). This indicates a significant public health risk, underscoring the need for stringent regulatory measures.

The bromate levels in 10 pizza and 12 naan samples collected from various locations in Dhaka city show significant variation (Table 5). The pizza samples had bromate concentrations ranging from 0.92 to 4.35 mg kg^−1^. The highest bromate concentration was detected in a pizza sample from Dhanmondi (road 8) (4.35 mg kg^−1^), while the lowest was from Shyamoli (0.92 mg kg^−1^). The RSD for pizza samples ranged from 2.44% to 13.12%, indicating varying degrees of measurement precision, with some samples showing notable variability. The analysis of pizza samples from Dhaka city revealed an average potassium bromate concentration of 2.19 mg kg^−1^. KBrO_3_ was found in 100% of the samples, all exceeding the FDA's permissible limit of 0.02 mg kg^−1^. The contamination levels ranged from 46.00 to 217.50 times the permissible limit, with an average excess of 109.30 times (Figure 4). This substantial contamination indicates a serious public health risk, emphasizing the urgent need for regulatory intervention.

**TABLE 5:** Bromate in different pizza and naan samples collected from Dhaka city.

Pizza samples (*n* = 10)					Naan samples (*n* = 12)				
Pizza samples	Sampling date	Area of collection	Bromate (mg kg^−1^)	RSD (%)	Naan samples	Sampling date	Area of collection	Bromate (mg kg^−1^)	RSD (%)
P‐1	03.08.22	Dhanmondi (Road 8)	4.35 ± 0.01	2.46	N‐1	14.04.22	Shahbag	1.04 ± 0.01	21.92
P‐2	03.08.22	Dhanmondi (Road 10)	1.52 ± 0.01	9.85	N‐2	21.04.22	Mogbazar	1.53 ± 0.01	9.33
P‐3	07.08.22	Lalbagh	2.01 ± 0.01	2.44	N‐3	08.05.22	Anandabazar	0.93 ± 0.01	20.33
P‐4	10.08.22	Abdul Hasnat Road	2.2 ± 10.01	3.22	N‐4	19.05.22	Jatrabari	1.31 ± 0.01	4.91
P‐5	14.08.22	Lalbagh	1.77 ± 0.01	3.86	N‐5	02.06.22	Mirpur	0.85 ± 0.01	14.56
P‐6	16.08.22	Hossaini Dalan Road	2.52 ± 0.01	4.48	N‐6	13.06.22	Mohammadpur	1.85 ± 0.03	16.17
P‐7	16.08.22	Azimpur	2.77 ± 0.01	3.25	N‐7	29.06.22	Shyamoli	0.93 ± 0.01	16.60
P‐8	18.08.22	Satrowza	1.57 ± 0.01	5.13	N‐8	07.06.22	Savar	5.37 ± 0.02	3.47
P‐9	21.08.22	Dhanmondi (Road 12)	2.23 ± 0.01	3.74	N‐9	14.08.22	Uttara	BDL	—
P‐10	22.08.22	Shyamoli	0.92 ± 0.01	13.12	—	—	—	—	—

In contrast, the naan samples had bromate concentrations between 0.85 and 5.37 mg kg^−1^, with one sample from Uttara having bromate levels in BDL. The highest bromate level in naan was found in a sample from Savar (5.37 mg kg^−1^, sample N‐8). The RSD values for naan samples ranged from 3.47% to 21.92%, with particularly high variability in samples from Shahbag (21.92%) and Ananda bazar (20.33%), suggesting inconsistent bromate distribution or potential analytical challenges. The widespread detection of bromate in both pizza and naan samples raises significant food safety concerns, given bromate's classification as a potential carcinogen. The analysis of naan samples from Dhaka city showed an average potassium bromate concentration of 1.73 mg kg^−1^. KBrO_3_ was present in 88% of the samples, all of which exceeded the FDA's permissible limit of 0.02 mg kg^−1^. The contamination levels ranged from 42.50 to 268.50 times the permissible limit, with an average exceedance of 86.31 times (Figure 4). This significant contamination underscores a serious public health concern, necessitating prompt regulatory action.

The Bangladesh Standards and Testing Institution (BSTI) allows a maximum of 5 mg of potassium bromate per kilogram of bread (Mahmud 2021). In some nations, maximum acceptable levels have been set for finished baked goods: the United States allows up to 0.02 mg kg^−1^, Japan up to 10 mg kg^−1^, and China up to 50 mg kg^−1^ (Oyekunle et al. 2014; Kurokawa et al. 1990). Despite these bans and restrictions, potassium bromate continues to be used extensively in bread making (Elsheikh et al. 2016). The bakery sector must adhere to protocols and guidelines to reduce potential residues of bromate in baked products to a safe threshold determined by FDA risk assessment at 0.02 mg kg^−1^ (American Bakers Association and American Institute of Baking International (ABA/AIBI) 2008; Ekop, Obot, and Ikpatt 2008). The elevated levels of potassium bromate detected in this investigation could potentially stem from its overutilization, constrained baking temperatures, and abbreviated baking durations, facilitating the complete conversion of potassium bromate into a benign and less hazardous potassium bromide byproduct (Akunyili 2004). Typically, bakers may opt for heightened concentrations of potassium bromate due to its capacity to enhance the aesthetic appeal of bread to consumers (Mahmud et al. 2021). These findings underscore the importance of stringent quality control measures in the production of bakery products to minimize bromate levels and protect public health. Future research should focus on identifying the sources of bromate contamination and developing strategies to eliminate its presence in food products.

## 4 Conclusion

The research on the estimation of the potential carcinogen KBrO_3_ in baked goods across Bangladesh reveals concerning trends. Few studies have quantified KBrO_3_ in bread, and none have examined other baked products like cake, burger buns, pizza, and naan until now, except for this research in Bangladesh. The presence of KBrO_3_ in bread samples from all divisions, nationwide cake samples, and specific bakery products in Dhaka exceeded permissible limits, highlighting significant food safety issues. The findings emphasize the need for regulatory measures to protect public health. Immediate actions such as stringent monitoring of bakery processes, enforcement of quality control standards, and public awareness campaigns are crucial. Further research should focus on finding alternative additives or baking techniques that eliminate the need for KBrO_3_. Collaboration between government agencies, food manufacturers, and consumer advocacy groups is essential to implement effective strategies and ensure the safety of baked goods in Bangladesh. Proactive measures and a strong food safety culture can help reduce the prevalence of KBrO_3_ in baked goods and promote healthier food practices.

## Author Contributions

**Abida Sultana:** conceptualization (equal), funding acquisition (equal), investigation (equal), project administration (equal), resources (equal), supervision (equal), writing – review and editing (equal). **Md. Mazharul Islam:** writing – original draft (equal). **Supath Xavier Besra:** formal analysis (equal), investigation (equal), validation (equal), visualization (equal). **Shahnaz Akhtar Nishat:** formal analysis (equal), investigation (equal), methodology (equal), validation (equal).

## Ethics Statement

The authors have nothing to report.

## Conflicts of Interest

The authors declare no conflicts of interest.

## Data Availability

Data are available on request from the authors.
